# Glucose-6-phosphate Dehydrogenase (*G6PD*) A-Variant Frequency and Novel Polymorphism in Haiti

**DOI:** 10.4269/ajtmh.22-0375

**Published:** 2022-10-03

**Authors:** Jeanne P Vincent, Alexandre V Existe, Kanako Komaki-Yasuda, Jacques Boncy, Shigeyuki Kano

**Affiliations:** ^1^ National Center for Global Health and Medicine, Tokyo, Japan;; ^2^ Institut Pasteur, Paris, France;; ^3^ Laboratoire National de Santé Publique, Port-au-Prince, Haiti

## Abstract

There are scarce data about the glucose-6-phosphate dehydrogenase (*G6PD)* variants in Haiti to guide public health guidelines. In this study, we investigated the prevalence of the *G6PD* mutations related to the A- variant. We found an allelic frequency of 35.8% for the A376G mutation and of 12.2% for the G202A mutation. We also found a novel C370T mutation concomitant with the A376G mutation in one study participant. The G680T and T968C mutations were not found. The G6PD deficient variant A^202^ (A376G and G202A mutations) has appreciable prevalence in Haiti (16.6%), consideration is warranted when using drugs such as primaquine, which may trigger hemolytic anemia among G6PD-deficient people.

The deficiency of the enzyme glucose-6-phosphate dehydrogenase (G6PD), which follows a geographic prevalence pattern that has been related to malaria selection,^1^ is associated with hemolytic anemia. The antimalarial primaquine is among the agents that may trigger hemolysis among G6PD-deficient people, and primaquine-sensitive erythrocytes were crucial in demonstrating the pathophysiology of the G6PD deficiencies.^2^ Primaquine is currently used for radical cure of *Plasmodium vivax* and *Plasmodium ovale* malaria (0.25–0.50 mg base/kg for 14 days) and in a single dose (0.25 or 0.75 mg base/kg) as a gametocytocide to complement *Plasmodium falciparum* treatment.^3^ Seeking relief of the country’s malaria burden, the Haitian Ministry of Health updated the malaria treatment guidelines in 2012 to add a single dose (0.75 mg base/kg) of primaquine.^4^

Haiti is a Caribbean country with the majority of its population being of West African ancestry. Thus, the deficiency variants reported among Africans and African Americans are expected to be found among the Haitian population.^5^ Moreover, deficiency prevalence exceeding 10% have been reported among other Latin American and Caribbean countries,^6^ and a study in one of Haiti’s departments found that 33 of 168 patients carried the G6PD variant A376G/G202A.^7^ In this study, we investigated the frequencies of the G6PD A- variants A376G/G202A, A376G/G680T, and A376G/T968C in Haiti at the DNA level and also report a novel variant.

We conducted a malaria survey in 2018 and 2019 in several cities across Haiti.^8^ During this survey, the participants were tested by rapid diagnostic test (RDT) at the site of recruitment, and dried blood spots were prepared for subsequent analyses including a malaria diagnosis by nucleic acid testing such as a polymerase chain reaction and loop-mediated isothermal amplification, sequencing of *P. falciparum* chloroquine resistance transporter, and sequencing of the human G6PD gene. The patients with a positive RDT at the point of care were treated according to the country’s guidelines (chloroquine 25 mg/kg over 3 days + primaquine 0.75 mg in a single dose under direct observation on day 0). During the recruitment period, no case of hemolytic anemia after treatment was brought to the attention of the research staff who were working full time at the recruiting sites.

The G6PD gene was sequenced for a subset of the original study population spread across five Haitian departments. The participants recruited in Dondon (North department), Grand-Goâve (West department), and Port-Salut (South department) were automatically included, whereas a random list of numbers was generated to select between the higher number of participants from Les Anglais (South), Dame-Marie (Grand’Anse department), and Baradères (Nippes department). The following nucleotides related to the A- variants were targeted: 202 in complementary DNA (exon IV), 376 (exon V), 680 (exon VII), and 968 (exon IX); four types of amplicons were obtained to overlap the polymorphisms of interest. All 199 samples were analyzed for exons IV and V, whereas only 106 were analyzed for exons VII and IX. This study was approved by the Haitian National Bioethics Committee (Ref: 1718-41) and by the NCGM Institutional Review Board for Clinical Research (Ref: 3332).

The 199 participants had a median age of 29 years (interquartile range 15–44) and included 111 females (55.8%). We found an overall prevalence of variant A (A376G) of 47.2% and of variant A^202^ (A376G/G202A) of 16.6%, which represents nine hemizygous males, five homozygous females, and 19 heterozygous females (Table 1). This result is in line with the previous genetic report from Haiti, even though the population in the present study is more widespread over the country.^7^ In this analysis, we did not find the G680T mutation or the T968C polymorphism that appeared to be common (10%) in the Sereer ethnic group in Senegal,^9^ although it has been reported in the Caribbean and found to represent 7% of the deficiency variants from an Amazonian population in Brazil.^10^

**Table 1:** Prevalence of G6PD variants, Haiti

	Site						
	Baradères	Dondon	Grand-Goave	Port-Salut	Dame-Marie	Les Anglais	Total
Participants, *n*	50	20	19	37	35	38	199
A376G							
Allelic frequency (%)	33.3	41.9	39.4	28.6	39.6	37.3	35.8
Males hemizygous (*n*, %)	7/22 (31.8)	3/9 (33.3)	1/5 (20.0)	6/18 (33.3)	4/17 (23.5)	5/17 (29.4)	26/88 (29.5)
Females homozygous (*n*, %)	3/28 (10.7)	2/11 (18.2)	4/14 (28.6)	3/19 (15.8)	1/18 (5.6)	4/21 (19.0)	17/111 (15.3)
Females heterozygous (*n*, %)	13/28 (46.4)	6/11 (54.5)	4/14 (28.6)	4/19 (21.1)	15/18 (83.3)	9/21 (42.9)	51/111 (45.9)
A376G/G202A (A^202^-)							
Allelic frequency (%)	15.4	16.1	6.1	12.5	9.4	11.9	12.2
Males hemizygous (*n*, %)	4/22 (18.2)	2 /9 (22.2)	0/5 (0.0)	2/18 (11.1)	1/17 (5.9)	0/17 (0.0)	9/88 (10.2)
Females homozygous (*n*, %)	3/28 (10.7)	0/11 (0.0)	1/14 (7.1)	1/19 (5.3)	0/18 (0.0)	0/21 (0.0)	5/111 (4.5)
Females heterozygous (*n*, %)	2/28 (7.1)	3/11 (27.3)	0/14 (0.0)	3/19 (15.8)	4/18 (22.2)	7/21 (33.3)	19/111 (17.1)
A376G/C370T (novel)							
Allelic frequency (%)	0	0	0	0	0	1.7	0.3
Males hemizygous (*n*, %)	0/22 (0.0)	0/9 (0.0)	0/5 (0.0)	0/18 (0.0)	0/17 (0.0)	0/17 (0.0)	0/88 (0.0)
Females homozygous (*n*, %)	0/28 (0.0)	0/11 (0.0)	0/14 (0.0)	0/19 (0.0)	0/18 (0.0)	0/21 (0.0)	0/111 (0.0)
Females heterozygous (*n*, %)	0/28 (0.0)	0/11 (0.0)	0/14 (0.0)	0/19 (0.0)	0/18 (0.0)	1/21 (4.8)	1/111 (0.9)

One female participant presented a previously unknown mutation, C370T, for which she was heterozygous (Figure 1). This mutation induced a change from histidine to tyrosine at position 124 of the amino acid chain. The same participant was homozygous for the A376G mutation, which caused the change from asparagine to aspartic acid in the G6PD variant A at position 126. This study did not analyze phenotype; we lack information on the enzyme activity with this double mutation. A study in Sierra Leone has also reported a double mutation on exon V (G311A/A376G) in a boy with low residual enzyme activity.^11^ In our case, however, the participant being a heterozygous female would limit our conclusion about this novel variant. It would have helped to record the G6PD activity for her and her parents, but we could not recontact them. This participant had also tested negative for malaria and thus was not qualified to receive primaquine on that occasion.

**Figure 1.**
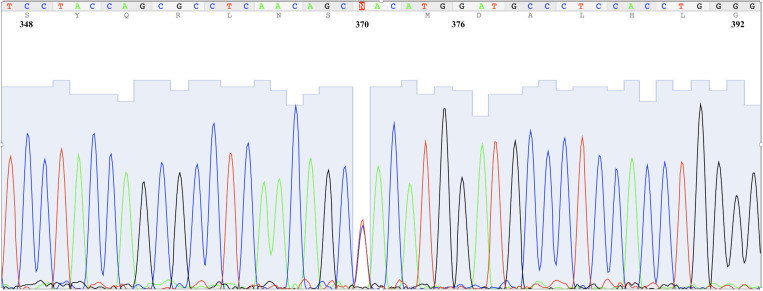
Electrophoregram of the novel Haiti variant. Position is according to complementary DNA nucleotides, isoform b (NCBI Reference Sequence: NM_001042351.3). C and T are seen at position 370 instead of T (heterozygous for this mutation); G is seen at position 376 instead of A (homozygous). This figure appears in color at www.ajtmh.org.

This study showed that the G6PD variants A and A^202^ were spread in Haiti. At approximately 10 years of implementation of the present policy in Haiti, no case of hemolysis after administration of the single dose of primaquine has been officially registered. We think this is due to the low probability of the appearance of a severe reaction after single-dose (0.75 mg/kg) primaquine among individuals with the A376G/G202A variant. In Vanuatu, the policy was discontinued after seven cases of acute hemoglobinuria with severe anemia were recorded over a 2-year period among G6PD-deficient men.^12^^,^^13^ The variants reported from Vanuatu were different from those of the population in the present study.^14^ The severity of the triggered hemolysis is variable, depending on G6PD variant and drug dosage, and it is subclinical in the majority of cases.^15^ In Haiti, moderate and mild reactions are more likely to be missed, especially if a patient does not seek formal medical care.

The WHO recommended the addition of primaquine at 0.75 mg base/kg (adult dose 45 mg) to treatment regimens for *P. falciparum* malaria in areas of low transmission, when the risk for G6PD deficiency was considered low or testing for deficiency was available. Because G6PD testing was not usually available at the point of care in malaria endemic regions, this limited the use of primaquine with some countries choosing to add it and some not.^15^ Considering safety concerns and to improve the uptake of primaquine, the WHO recommendation has been updated to a single dose of 0.25 mg base/kg, which should similarly reduce *P. falciparum* transmissibility and should not cause serious toxicity, even in people with G6PD deficiency.^16^ However, the policy was not updated in Haiti where single-dose primaquine (0.75 mg base/kg) is administered to malaria cases under direct observation without G6PD testing. The evidence to support the use of primaquine as gametocidal drug at low dose (0.25 mg base/kg) is particularly relevant when primaquine is combined with artemisinin-based combination therapy, which is not the case in Haiti (chloroquine is still used as first line).^13^

The findings of the present study show a considerable prevalence of G6PD variant A^202^ (16.6%) and indicate that there might be other less frequent polymorphisms occurring in Haiti that have not been revealed due to the scarcity of investigation. Consistent gametocytocidal effect at reduced risk among the G6PD-deficient people, using a low-dose primaquine regimen is an option that should be explored in a local trial considering local vectors and drug combinations.
